# The Impact of Sensory Reactivity and Oral Praxis on Feeding Participation in Children with Autism (SemAlTea Study)

**DOI:** 10.3390/bs15111577

**Published:** 2025-11-18

**Authors:** Inmaculada López-Martínez, Rafael Galera-Martínez, Adrián Aparicio-Mota, José María López-Martín, Isabelle Beaudry-Bellefeuille, Tesifón Parrón-Carreño

**Affiliations:** 1 Bimba Pediatric Clinic, Torrecárdenas University Hospital, 04009 Almería, Spain; clinicabimba@gmail.com; 2 Pediatric Gastroenterology and Nutrition Unit, Torrecárdenas University Hospital, 04009 Almería, Spain; 3 Biomedical Research Unit, Andalusian Public Foundation for Biomedical Research in Eastern Andalusia (FIBAO), Torrecárdeas University Hospital, 04009 Almería, Spain; 4 Beaudry-Bellefeuille Pediatric Occupational Therapy Clinic, 33007 Oviedo, Spain; ibbergo@gmail.com; 5 Department of Nursing, Physiotherapy, and Medicine, Faculty of Health Sciences, University of Almería, 04120 Almería, Spain

**Keywords:** autism, oral reactivity, tactile reactivity, avoidance, participation, feeding problems, oral praxis problems, parental stress

## Abstract

Clarifying the influence of sensorimotor factors on feeding participation problems (FPPs) in children with autism may have implications for therapeutic interventions. Our objective was to assess the prevalence of FPPs in a population of children with autism and to study its association with sensorimotor factors (oral and tactile reactivity and oral praxis). Descriptive observational study including 26 autistic children aged 3 to 12 years. Behavioral Pediatrics Feeding Assessment Scale (BPFAS), Sensory Profile 2 (SP-2), and Oral Praxis (OPr) tests were used. Caregiver stress was evaluated by Parental Stress Index–Short Form (PSI-4-SF). Multivariate logistic analysis and Receiver Operating Characteristic curve was used to predict the risk of FPPs. 26 children (84.6% boys) were included, with a mean age of 6.92 years (95% CI 5.94–7.91). Feeding behavior difficulties were observed in 53.8% of participants, 80.7% exhibited oral praxis issues, and 65.4% of caregivers reported stress related to their child’s FPPs. Oral reactivity and sensory avoidance behaviors were significantly associated with FPPs. Among children with higher support needs, tactile reactivity was also significantly related to FPPs. FPPs in children with autism were associated with oral and tactile hyperreactivity, higher levels of sensory avoidance, dysfunctional parent–child interactions, and increased support needs.

## 1. Introduction

The increasing prevalence of autism spectrum disorder (ASD) diagnoses, estimated to be around 1/100 (Maenner, 2020; Zeidan et al., 2022), along with the current demand for treatments and therapeutic approaches, exponentially increased the interest and importance of carrying out family and child-centered interventions in social, school, and family settings. Children with ASD display differences in sensory reactivity, sensory perception, and sensory-based praxis abilities (Roley et al., 2015; Schulz & Stevenson, 2019). Sensory differences may exist for both environmental stimuli and body sensations (Baranek et al., 2014; Posar & Visconti, 2018). Such issues may lead to feeding participation problems (FPPs), a frequent reason for occupational therapy consultation (Reche-Olmedo et al., 2021). FPPs are frequent in children with ASD, linked to a variety of factors and manifestations such as food rejection, texture tolerance, oral-motor skills, swallowing ability, and mealtime participation issues (Beaudry-Bellefeuille et al., 2021; Chistol et al., 2018). All this can generate stress in the primary caregivers and negatively impact the nutritional status of the children (Curtin et al., 2015).

Our primary objective was to analyze FPPs in a population of children with ASD and to study their association with sensorimotor factors (reactivity, praxis). Secondary objectives included assessment of the impact of FPPs on the nutritional status of the children and on the stress level and coping strategies of the caregivers.

## 2. Materials and Methods

This was an observational study conducted prospectively between January 2020 and December 2020. The accessible study population consisted of children with a diagnosis of ASD enrolled in classrooms for autistic children in the metropolitan area of Almería (Spain). Inclusion criteria were children aged between 3 and 12 years old with a diagnosis of ASD, according to DSM-V criteria (American Psychiatric Association, 2013). Children with comorbidity with genetic syndromes (such as Rett syndrome), severe sensory disturbances (such as blindness or deafness), or a language barrier with the family, were excluded.

For recruitment, the education delegation of the area was contacted to request access to the population in classrooms for autistic children in the city. The autism classroom coordinators were informed of the research project in order to offer participation to the families of autistic children. The principal investigator (PI) scheduled appointments with the schools to inform the families about the project and provided them with a project information sheet. Subjects whose families were interested and informed of the study procedure by the PI were recruited. The evaluations were carried out at the child’s school or at the child’s therapy clinic, according to family preference. The parents who were actively involved in their child’s feeding completed the questionnaires in the presence of the PI to clarify any doubts concerning the items.

### 2.1. Study Variables and Evaluation Tools

#### 2.1.1. Socio-Demographic Variables

Socio-demographic data were collected from the caregivers of the participants, including age, sex, schooling modality, and attendance to therapies. Socioeconomic status of parents was classified according to the European Socioeconomic Classification (ESeC) (Harrison & Rose, 2006). ESeC is a categorical social class classification based on employment status. Its objective is to determine different positions within the labor market and production units in terms of typical “labor relations.” The ESeC establishes 10 classes, which can be grouped into 3 main classes (Class 1: Managers and professionals; Class 2: Intermediate occupations; and Class 3: Working class), to which an extra class was added to include unemployed workers.

#### 2.1.2. Classification of Autism Spectrum Disorder

In accordance with DSM-V diagnostic criteria, the level of severity of the ASD diagnosis of each child was documented based on the type of help the person needed to function at home, school, and other vital contexts: Level 1 = requires support; Level 2 = requires substantial support; and Level 3 = requires very substantial support (American Psychiatric Association, 2013).

#### 2.1.3. Feeding Participation

The main variable in this study was feeding participation measured via the Behavioral Pediatrics Feeding Assessment Scale (BPFAS) (Crist & Napier-Phillips, 2001), consisting of 35 items designed entirely to assess the feeding-related behaviors of children aged 9 months to 12 years, as well as parental and filial behaviors associated with poor nutritional intake. The BPFAS was previously validated in children with ASD (Allen et al., 2018). In this assessment, two scores are obtained: the frequency score (FS), which measures difficulties in eating behavior and thus the risk for an eating disorder, and the problem score (PS), related to problems with feelings or strategies for coping with feeding difficulties on the part of families. Both scores obtained are compared with a standardized reference mean, and a score is considered pathological when the value obtained is higher than 84 points for the FS and higher than 9 points for the PS.

#### 2.1.4. Sensorimotor Factors

For the analysis of sensory reactivity, the Sensory Profile 2 (SP-2) questionnaire (Dunn, 1999; Romero-Ayuso et al., 2016) was used, and for the study of oral-motor praxis, the Oral Praxis (OPr) test of the Sensory Integration and Praxis Tests (SIPT; Ayres, 1989) was used. The SP-2 is a caregiver questionnaire that provides information concerning the child’s behavior in response to sensory input in daily life situations. The SP-2 provides a quadrant score based on items representing different sensory systems, with the items grouped as categories of response to the sensory-laden situations: seeking, avoiding, sensitivity, and registration. The SP-2 also offers scores by sensory systems (auditory, visual, touch, movement, body position, oral). The items included in each sensory section of the SP-2 explore hyper- and hypo-reactivity to sensation.

To assess oral praxis skills, the OPr test (Ayres, 1989) was used. The OPr test is a performance measure administered by a trained examiner in which the child is asked to imitate oral movements. Scores are based on the quality of coordination and sequencing of the participant’s jaw, cheeks, lips, and tongue movements.

#### 2.1.5. Anthropometry

Anthropometry was performed according to standardized methodology (Ros Arnal et al., 2011) on all patients by assessing their weight (kg) and height (cm) using a portable digital scale with a Soehnle Professional measuring rod with an accuracy ±100 g and ±1 cm. Body mass index (BMI) was calculated as kg/m^2^. Z-scores were calculated using the World Health Organization (WHO) growth standards for weight, height, and BMI (De Onis et al., 2006; De Onis et al., 2007). Based on the BMI Z-score, patients were classified as obese (>+2 standard deviation [SD]), overweight (>+1 SD), normal (between −1 and +1 SD), thin (<−1 SD), or undernourished (<−2 SD) (Ros Arnal et al., 2011).

Arm circumference (cm) and abdominal circumference (cm) were measured using an inextensible tape measure. Arm circumference Z-scores were calculated using WHO standards (De Onis et al., 2006) for children younger than 5 years and the enKid Study (Serra Majem et al., 2002) for those older than 5 years. Abdominal circumference Z-scores were calculated using the enKid Study, and abdominal obesity was defined as the Z-score >+2 SD (Serra Majem et al., 2002).

#### 2.1.6. Caregiver Stress

The Parental Stress Index (4th ed.), Short Form (PSI-4-SF) (Abidin, 2012) was used to assess caregiver stress, classifying caregiver stress into three independent subscales and a total stress score called parental distress (PD), dysfunctional parent–child interaction (P-CDI), difficult child (DC), and total stress (TS).

### 2.2. Sample Size Calculation

As there were no previous studies analyzing the correlation between scales measuring feeding participation and sensory-motor factors in children with ASD, a correlation of 0.60 between the BPFAS and SP-2 was considered relevant. Likewise, a power of 90% was considered to detect differences in the contrast of the null hypothesis (H_0_: ρ = 0) by means of a bilateral Student’s *t*-test for the Pearson’s correlation coefficient between two variables. Considering that the significance level is 0.05, it was necessary to include 21 children in the study. The sample size calculation program Ene 3.0 was used for this calculation.

### 2.3. Statistical Methods

Qualitative variables (BPFAS, SP-2, and PSI-4-SF) were expressed as percentages, and group differences were assessed using the chi-squared test. Quantitative data (Z-scores, OPr test) were described by mean and SD. The Kolmogorov–Smirnov test was used to determine whether the analytical variables were normally distributed. For parametric variables, the Student’s *t*-test was used to compare means, and in the case of nonparametric variables, the Mann–Whitney U test. A *p*-value of less than 0.05 was considered significant. To estimate the risk of FPP, we fitted a multivariable logistic regression. Candidate predictors were prespecified based on clinical relevance; additionally, variables with *p* < 0.15 in bivariable analyses were considered. Age and sex were included a priori as potential confounders regardless of bivariable significance. Variable selection used backward elimination retaining variables with *p* < 0.10 and clinical plausibility. IBM’s SPSS (Version 25) software was used for these calculations.

## 3. Results

A total of 26 children (84.6% boys) were included, with a mean age of 6.92 years (95% CI 5.94–7.91) and a range between 3 and 12 years. In relation to the severity of the ASD diagnosis, 7.7% needed support (Level 1), 30.8% needed substantial support (Level 2), and 61.5% needed very substantial support (Level 3). Most (80.8%) of the children were enrolled in special classrooms for autistic children, and the rest attended regular classrooms with support. Most of them attended therapies outside of school on a regular basis: 84.6% occupational therapy, 88.5% some psychological service, 84.6% speech therapy, and 15.4% music therapy. In addition, 26.9% attended other activities like swimming. Regarding socioeconomic status, a higher percentage of unemployed mothers (26.9%) was observed compared to 3.8% in the case of fathers. However, the percentages of mothers and fathers who worked at level 1 (managers and professionals) and level 2 (intermediate occupations) were similar.

### 3.1. Participation in Feeding

In relation to the feeding participation assessed with the BPFAS, the mean FS was 85.19 (95% CI 79.68–90.71) and the PS was 13.15 (95% CI 9.88–16.43). Most (53.8%) of the children presented difficulties in feeding behavior (pathological FP), and 65.4% of the families reported problems with feelings or strategies to cope with the feeding difficulties of their child (pathological PS). There was no association between feeding participation problems and the socio-economic level of either parent.

### 3.2. Oral Praxis

The OPr test was administered to all participants, with a mean score of −2.24, ranging from 0.96 to −3.00. On this test, 21 of the 26 participants (80.7%) fell below 1 SD of the normative sample’s mean and were therefore considered to have oral praxis problems. Children with autism have oral praxis problems, but we did not see that this was significantly associated with feeding problems.

### 3.3. Sensory Reactivity (SP-2)

Regarding SP-2 results, considering as pathological all scores included in both “more than others” and “much more than others” categories, 80.8% showed avoidance, 92.3% showed sensitivity, 73.1% had problems with touch processing, and 76.9% had problems with oral processing. The children in the pathological BPFAS FS group had a higher SP-2 oral section score than those in the non-pathological BPFAS FS group (Table 1), which reflects more sensory problems in the pathological BPFAS FS group. In addition, children in the pathological BPFAS PS group showed higher SP-2 oral section and SP-2 avoidance quadrant scores than the non-pathological BPFAS PS patients (Table 1).

When we analyzed only those patients with an ASD grade 2 or higher, we found that those in the pathological BPFAS PS group, in addition to having higher scores on the SP2 oral section than those in the non-pathological BPFAS PS group, also showed a significantly higher score in the avoidance and tactile sections (Table 2).

### 3.4. Anthropometry

A minority (3.8%) of the patients were undernourished, 23.1% were overweight, 23.1% were obese, and 19.2% had abdominal obesity. Most (57.7%) of the patients had a normal arm circumference Z-score, 15.4% < −1 SD and 26.9% > 1 SD. No significant differences were found in the Z-score for BMI, BW, and BP, or in the prevalence of undernutrition, overweight, obesity, or abdominal obesity among the children according to whether or not they had eating behavior difficulties or whether families reported problems with feelings or coping strategies for eating difficulties.

### 3.5. Caregiver Stress (PSI-4-SF)

**Figure 1:**
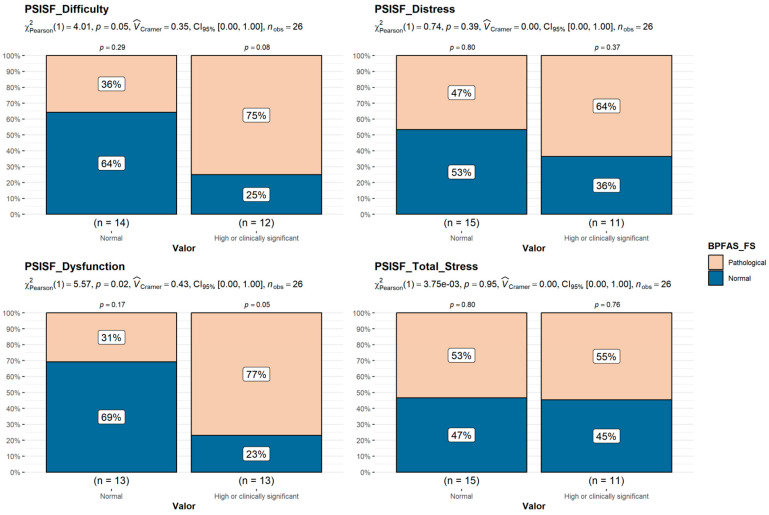
Comparison of BPFAS Frequency Score (FS) in the “high or clinically significant PSI-SF” groups vs. normal range groups (Chi-squared test).

**Figure 2:**
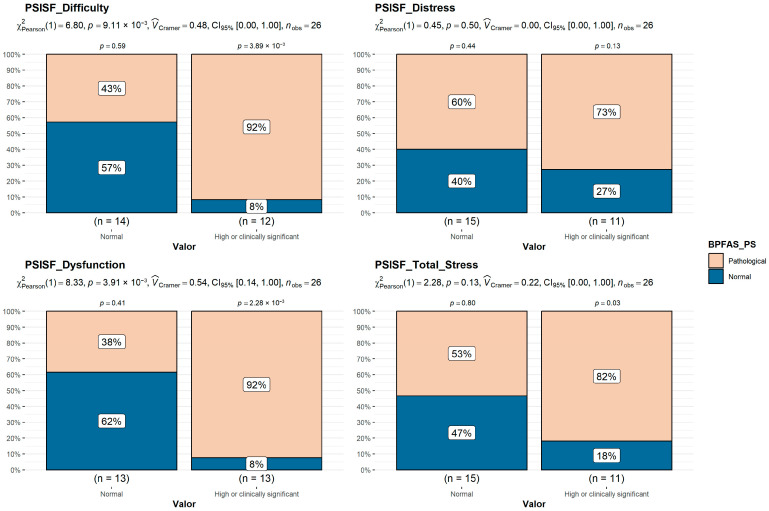
Comparison of BPFAS Problem Score (PS) in the “high or clinically significant PSI-SF” groups vs. normal range groups (Chi-squared test).

Some (38.5%) of the parents presented “clinically significant stress” on the TS, PD, and P-CDI scales, and 30.8% of the parents presented “clinically significant stress” on the DC scale. Families whose children were in the pathological BPFAS FS or PS groups reported a significantly higher frequency of caregiver–child relationship dysfunction and greater child developmental difficulty (Figure 1 and Figure 2).

## 4. Discussion

The SemAlTea Study found a high prevalence of FPPs in children with ASD and a significant association between these FPPs and the oral sensory section profile and avoidance quadrant. Our FFPs prevalence is consistent with Zlomke et al. (2020), where more than 50% of their sample presented clinical difficulties in any of the child’s feeding behaviors (59.4%) and in the parents’ coping strategies (51.6%). Other authors (e.g., Kang et al., 2022) detected lower figures in an Asian population: 28.4% of the participants presented FPPs, using the total BPFAS scores. This difference can probably be explained in part by the cultural differences described in Kang et al.’s study population, who presented hyperactivity and experienced an authoritarian feeding style facilitated by parents. Other studies reported that feeding difficulties using the BPFAS develop more frequently in children with ASD at younger ages (15–36 months), thus running the risk of social and health consequences in young children with ASD (Ashley et al., 2020). These data along with the Şahan et al. (2021) study results solidify that feeding problems in the ASD population are significant, per BPFAS results. The percentages of the SemAlTea Study are somewhat lower possibly because the study participants were already attending therapies. Finally, Baraskewich et al.’s (2021) review indicates that 91% of the articles exploring autistic populations reference some kind of feeding problem.

Regarding the sensory factors in children with ASD, 73.1% of our population presented total pathological scores in the tactile sensory section and 76.9% in the oral sensory section of the SP-2; data consistent with Mayes and Zickgraf (2019), where 70.4% of the children with ASD presented atypical eating behaviors, food selectivity, and hypersensitivity to textures during a standardized interview. Leader et al. (2020) found high rates of gastrointestinal symptoms, challenging behaviors, sensory problems, food selectivity, and refusals to eat in an ASD population who obtained a mean of 23 in tactile sensitivity using the SP-2 short form, similar to the mean of 26.12 in the Sem AlTea Study participants with tactile reactivity.

The SP-2 SemAlTea Study population scores indicated sensory integration problems in a high percentage of the group. Kirby et al. (2019) concluded that the sensory characteristics of children with ASD can affect the everyday experiences of these children and their caregivers, for example, during feeding. This affects both the children themselves and their family’s environment, which is why more and more therapeutic options and an analysis of the difficulties are being sought to be able to apply specific treatments and improve areas of daily living, such as feeding.

Regarding the association between FPPs with sensory factors (reactivity, praxis), the mean score on the OPr test of the SIPT was −2.24, this score was pathological and indicative of a problem of oral praxis. Similarly, Roley et al. (2015) evaluated the sensory integration and praxis (with the SIPT) in an ASD population aged between 4 and 11 years, obtaining a mean score of −1.80, which was also pathological. However, we determined these data did not appear significantly associated with feeding problems. The difference in our results may be due to the degree of ASD of the children in our study, where 61.5% needed very noticeable help, evincing a relatively worse developmental level. Likewise, our data from the OPr and SP-2 assessments agree with what has been described by other authors on eating behavior disorders in children with ASD, including food refusal, texture tolerance problems (reactivity), and difficulties in taking part in the feeding process (participation) (Ismael et al., 2018).

In relation to nutritional status, FFPs were not associated with a higher prevalence of malnutrition in our study. A possible explanation is the food selectivity that children with ASD show towards caloric foods (carbohydrates and processed foods), which can partly compensate for the deficit in intake (Cermak et al., 2010; Mayes & Zickgraf, 2019), which also highlights the fact that 46.2% of patients presented overweight and obesity, and 19.2% abdominal obesity. However, children with difficulties in eating behavior tended to have lower anthropometric parameter means, although without reaching statistical significance. Since the sample size was not designed to detect these differences, it is possible that an increase in sample size would reflect variations in anthropometry. It should be noted that no information was collected on the children’s intake, nor were analytical determinations of micronutrients performed, which could have detected other types of nutritional problems.

Overall, the SemAlTea Study shows the significance between oral reactivity and the BPFAS FP (i.e., frequency score) and oral reactivity and the BPFAS PP (i.e., problem score). In the study population with ASD grades 2 and 3, we also found significance between tactile reactivity and PP results from the BPFAS. No other studies were found with autistic populations where both the BPFAS and SP-2 tests were applied to study FPPs. However, Kim et al. (2021) related feeding problems per the BPFAS and sensory problems in children with feeding refusal and concluded that addressing the senses improves feeding interventions.

Our study demonstrated a significance between PSI-4-SF parent–child interaction dysfunction and the BPFAS PS and PSI-4-SF child difficulty ratings with the BPFAS PS. Other authors have used the PSI-4-SF to assess parental stress in autistic populations to demonstrate the efficacy of physical activity programs (Zhao et al., 2021), but not in relation to feeding problems, thus no studies like the SemAlTea Study can be found.

Overall, our analysis of factors related to feeding in the ASD population has aimed to increase the understanding of people with autism per their daily feeding activity. We sought to provide data for assessment and therapeutic interventions that might improve their nutritional status and participation in occupational therapy, psychological services, and medical programs (Gándara-Gafo et al., 2021; Goday et al., 2019).

### 4.1. Limitations

Our study sample was composed of volunteers, which may have included families who were relatively more motivated or more concerned about FPPs. Conversely, given that a large proportion of the population was undergoing therapy, the prevalence of FPPs may have been influenced by the intervention they were receiving. Furthermore, the participants were recruited from a single center and shared a relatively homogeneous cultural background, which could bias their perceptions regarding their child’s FPPs, particularly when the assessments involve a certain degree of subjectivity. Although participant’s results were compared with the normative values of tests derived from neurotypical individuals, the absence of a control group in this study constitutes an additional limitation. Future research involving a larger and more culturally diverse sample would enhance the statistical power of future investigations examining similar associations and correlations.

### 4.2. Implications for Therapy Interventions

- Occupational therapy interventions for children with ASD who experience difficulties participating in feeding and mealtimes, that consider sensory integration problems of the tactile and oral systems, can be useful for improving the processing of these stimuli and the ability of the child with ASD to touch, explore, and eat food.
- Improvements in the underlying sensory factors and basic skills linked to feeding can lead to improvements in eating behaviors and mealtime participation, thus reducing the risk of suffering from an eating disorder, reducing caregiver stress, and improving the parent–child relationship.
- Larger studies are needed to confirm our results. Until then, if practitioners choose to implement this approach clinically, they need to carefully document treatment content, client responses to the treatment, and changes in client functioning (or occupational engagement) from the start to the termination of treatment.

## 5. Conclusions

The SemAlTea Study shows that FPPs in children with ASD are associated with greater oral sensory impairment and a more prominent sensory avoidance profile. Moreover, in those children with ASD grades 2 or 3, they are associated with greater tactile system sensory impairment. Also, FPPs were associated with greater dysfunction and interaction between the child and their parents and greater difficulties for the child.

## Figures and Tables

**Table 1:** Comparison of SP-2 and BPFAS test values in the total sample (N = 26).

	BPFAS FS Pathological			BPFAS PS Pathological		
	No	Yes	*p*	No	Yes	*p*
SP-2 Sensitivity	52.25	59.50	0.118	51.11	58.82	0.095
SP-2 Avoidance	48.50	55.07	0.193	45.33	55.59	0.013 *
SP-2 Oral	24.58	34.29	0.006 *	22.33	33.76	0.001 *
SP-2 Tactile	23.17	28.64	0.118	21.89	28.35	0.051
SP-2 Behavioral	21.92	26.14	0.212	22.33	25.18	0.494

* = Mann–Whitney U test. FS = frequency score on the BPFAS questionnaire; the cut-off point (pathological) = 84 points. PS on the BPFAS questionnaire was the problem score; the cut-off point (pathological) = 9 points.

**Table 2:** Comparison of SP-2 and BPFAS test values in ASD grade 2 and 3 Children (N = 24).

	BPFAS FS Pathological			BPFAS PS Pathological		
	Mean No	Mean Yes	*p*	Mean No	Mean Yes	*p*
SP-2 Sensitivity	52.82	57.54	0.277	51.11	57.93	0.084
SP-2 Avoidance	48.09	53.154	0.252	45.33	54.13	0.021 *
SP-2 Oral	23.91	33.54	0.007 *	22.33	33.20	0.002 *
SP-2 Tactile	24.18	28.15	0.277	21.89	29.00	0.03 *
SP-2 Behavioral	22.73	25.00	0.494	22.33	24.03	0.238

* = Mann–Whitney U test. FS = frequency score on the BPFAS questionnaire; cut-off point = 84 points. PS = BPFAS questionnaire problem score; cut-off point = 9 points.

## Data Availability

The data presented in this study are not publicly available due to privacy and ethical restrictions. Access to the data may compromise the confidentiality of participants and is therefore restricted.

## Abbreviations

The following abbreviations are used in this manuscript:

ASD	Autism Spectrum Disorder
FPPs	Feeding Participation Problems
BPFAS	Behavioral Pediatrics Feeding Assessment Scale
FS	Frequency Score
PS	Problem Score
SP	Sensory Profile 2
OPr	Oral Praxis
SIPT	Sensory Integration and Praxis Tests
PSI-4-SF	Parental Stress Index–Short Form
BMI	Body Mass Index
ROC	Receiver Operating Characteristic
ESeC	European Socio-Economic Classification
